# Association Between Echocardiographic Non-invasive Myocardial Work Indices and Myocardial Fibrosis in Patients With Dilated Cardiomyopathy

**DOI:** 10.3389/fcvm.2021.704251

**Published:** 2021-08-16

**Authors:** Cunying Cui, Yanan Li, Yuanyuan Liu, Danqing Huang, Yanbin Hu, Ying Wang, Lijia Ma, Lin Liu

**Affiliations:** ^1^Department of Ultrasound, The People's Hospital of Zhengzhou University, Henan Provincial People's Hospital, Fuwai Central China Cardiovascular Hospital, Zhengzhou, China; ^2^Department of Radiology, The People's Hospital of Zhengzhou University, Henan Provincial People's Hospital, Zhengzhou, China

**Keywords:** dilated cardiomyopathy, myocardial work, left ventricular systolic function, myocardial fibrosis, magnetic resonance imaging, echocardiography

## Abstract

**Objectives:** To analyze the association between global myocardial work indices evaluated by non-invasive left ventricular (LV) pressure-strain loop (PSL) and LV myocardial fibrosis in patients with dilated cardiomyopathy (DCM).

**Methods:** A total of 57 patients with DCM were included in this prospective study. Global work index (GWI), global constructive work (GCW), global wasted work (GWW), global work efficiency (GWE) and global longitudinal strain (GLS) were measured using LVPSL. LV volumes and LV ejection fraction (LVEF) were evaluated using cardiac magnetic resonance imaging (CMRI), LV myocardial fibrosis was estimated at CMRI by qualitative assessment of late gadolinium enhancement (LGE). According to the CMRI, the studied population was divided into two groups, namely: patients without LGE (LGE-) and patients with LGE (LGE+).

**Results:** The LGE+ group presented with increased age, LV end systolic volume (LVESV) index and reduced GWI, GCW, GWE, GLS, CMRI-derived LVEF (LVEF_CMRI_), the differences between the two groups were statistically significant (*P* < 0.05). After correcting for age and LVESV index, LVEF_CMRI_, GLS, GWI, GCW, and GWE retained independent associations with LV myocardial fibrosis. According to receiver operating characteristics (ROC) analysis, LVEF_CMRI_, and GCW showed larger AUC and higher accuracy, sensitivity, and specificity than GLS, the accuracy of predicting LV myocardial fibrosis ranged from high to low as: LVEF_CMRI_, GCW, GWE, GWI, and GLS.

**Conclusions:** LVEF_CMRI_, GWI, GCW, GWE, and GLS remained significant predictors of LV myocardial fibrosis. LVEF_CMRI_, and GCW appeared to better predict LV myocardial fibrosis compared with GLS.

## Introduction

Dilated cardiomyopathy (DCM) is defined as the dilatation and dysfunction of one or both ventricles in the absence of abnormal loading or coronary heart disease (CHD) (1, 2). This disease can occur in all age groups but is more common in young adults. The incidence rate in men is higher than that in women (3). With progression of the disease, it can lead to various complications, such as heart failure, shock, arrhythmia, and even sudden death (4). Accurate assessment of left ventricular function in DCM patients is extremely important for clinical diagnosis, treatment, and prognosis (5).

Cardiac magnetic resonance imaging (CMRI) is the gold standard for the measurement of cardiac function parameters (6, 7). In addition, the application of late gadolinium enhancement (LGE) is currently the most reliable method for non-invasive detection of localized myocardial fibrosis, which is helpful for the diagnosis of DCM and significantly related to its prognosis (8, 9). However, CMRI takes longer and is expensive, it is difficult to be used as a routine examination in clinical practice.

The left ventricular ejection fraction (LVEF) and global longitudinal strain (GLS) measured using echocardiography are often recommended for the evaluation of left ventricular systolic function (LVSF) (10). However, LVEF and GLS are susceptible to cardiac load. The myocardial work index derived from the left ventricular pressure-strain loop (LVPSL) is a new method for non-invasive assessment of LVSF (11). This technique is derived from the 2D speckle tracking technique, and it considers the effect of afterload on strain. The method is simple, easy, and non-invasive, and it could be widely used in clinical practice.

Previous studies by Chan et al. and the author confirmed the feasibility of the myocardial work index to evaluate LVSF in patients with DCM (12, 13). The present study aimed to analyze the correlation between global myocardial work indices and LV myocardial fibrosis in patients with DCM.

## Materials and Methods

### Study Population

**Figure 1:**
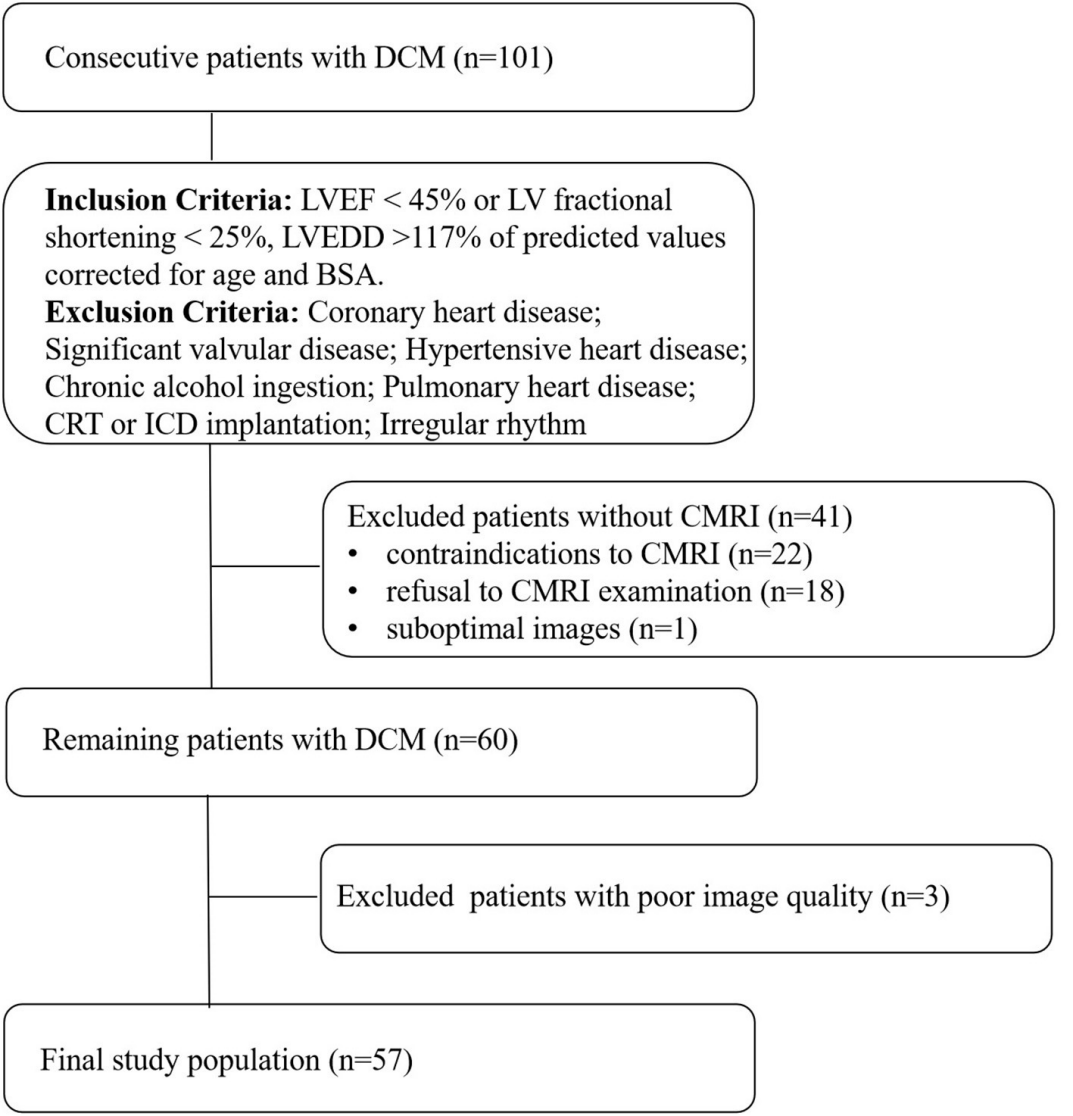
Flow chart detailing the identification of the study cohort. Contraindications to CMRI include incompatible metallic devices, contrast medium allergy, or claustrophobia. Significant valvular disease was defined as valvular stenosis or > mild functional regurgitation. Functional mitral regurgitation was secondary to left ventricular remodeling, mitral valve anatomy was normal. DCM, dilated cardiomyopathy, LVEF, left ventricular ejection fraction, LVEDD, left ventricular end-diastolic diameter, BSA, body surface area, CMRI, cardiac magnetic resonance imaging, CRT, cardiac resynchronisation therapy, ICD, implantable cardioverter defibrillator.

We conducted a prospective study on 101 consecutive patients with DCM in the heart failure department of Fuwai Central China Cardiovascular Hospital from January 2019 to February 2021. The diagnosis of DCM was established according to current guidelines (14). The inclusion criteria were: (1) LV ejection fraction (LVEF) ≤ 45% or LV fractional shortening <25%; (2) LV end-diastolic diameter > 117% of predicted values corrected for age and body surface area (BSA), both echo-determined. Patients with coronary heart disease (> 50% angiographical stenosis in any epicardial coronary artery, and patients with an ischemic scar at cardiac MRI), hypertensive heart disease, significant valvular disease (valvular stenosis or > mild functional regurgitation), chronic alcohol ingestion, pulmonary heart disease, congenital heart disease, a history of cardiac resynchronisation therapy (CRT) and implantable cardioverter defibrillator (ICD) implantation, or irregular rhythm were excluded. The 41 patients who had not undergone a CMRI were excluded. Finally, 3 patients were excluded from this study because of poor image quality. The final analysis included 57 DCM patients (Figure 1).

We recorded medical history, including New York Heart Association (NYHA) functional class; biomarkers; cardiovascular risk factors; current medications and 12-lead electrocardiography (ECG). ECG, Transthoracic echocardiography and CMRI were performed within 24 h.

### Transthoracic Echocardiography

All echocardiographic examinations were performed on a Vivid E95 ultrasound system (GE Vingmed Ultrasound AS, Horten, Norway) equipped with an M5Sc-D 1.4–4.6 MHz transducer. All the study subjects were placed in a left-side position and synchronously connected to the electrocardiogram. The average frame rate of the 2D image was 59 ± 7 frames/sec. The LVEF of each patient was measured using the biplane Simpson method. The peak velocity of the mitral valve in early diastolic period (E peak), and the average velocity of the mitral annulus (e′) were measured, and E/e′ was calculated. Apical four-, three-, and two-chamber images were continuously acquired for at least three cardiac cycles, and the Doppler blood flow spectrum of the aortic and mitral valves was obtained. The original data images were saved to a hard disk for analysis.

### Image Analysis for Myocardial Work

**Figure 2:**
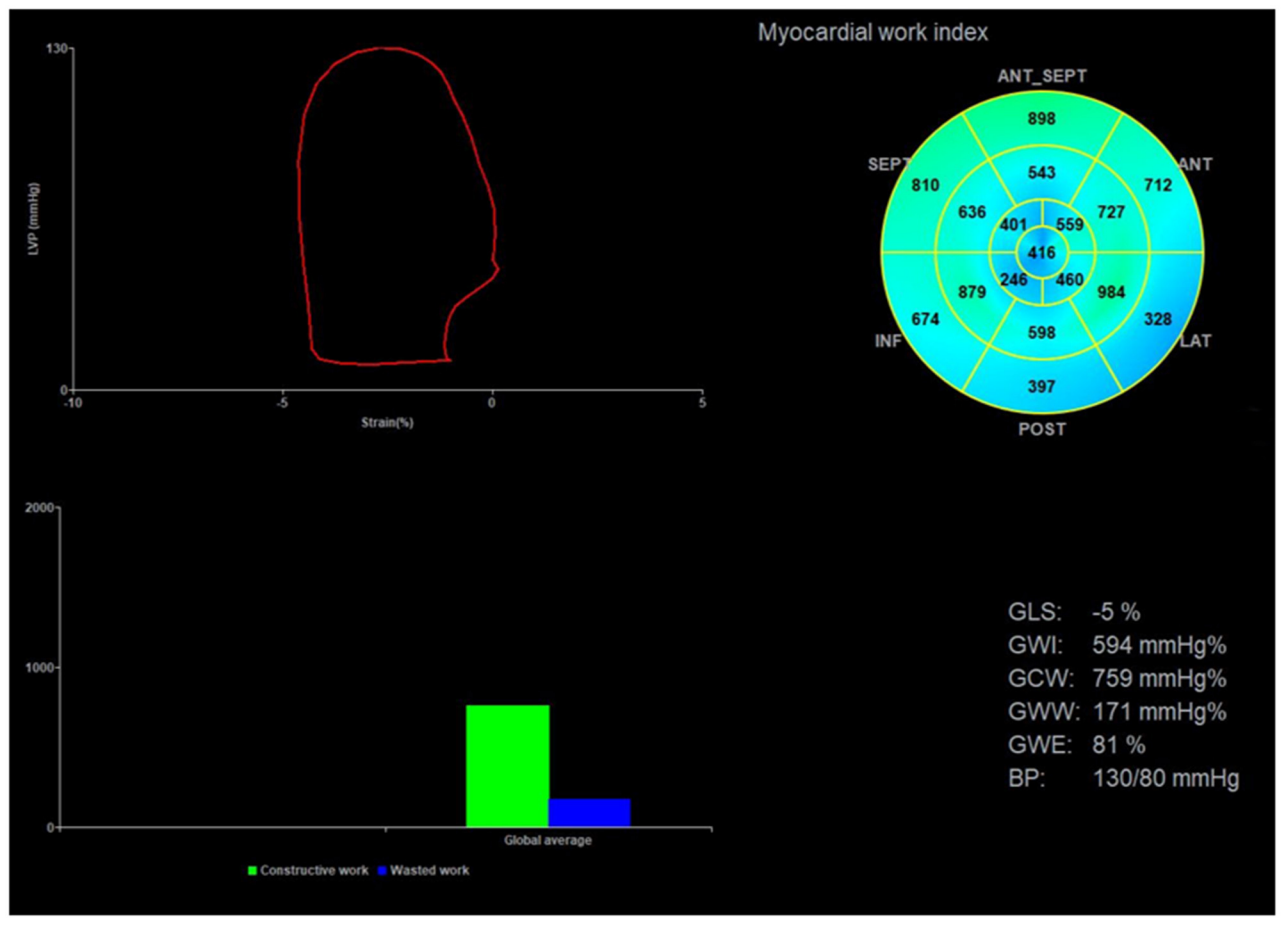
Image analysis for myocardial work. The upper left corner of the image is the left ventricular PSL curve, the upper right corner is the 17-segment myocardial work index bull's eye diagram, and the lower right corner displays the left ventricular global myocardial work indices obtained from the PSL curve. PSL, pressure-strain loop.

Echopac version 203 (GE Vingmed Ultrasound AS, Horten, Norway) was used for image analysis. According to the Doppler flow spectrum of the aortic valve and mitral valve, valvular event time was determined. Tracking was automatic, but if the tracking was not satisfactory, tracking points were adjusted manually to determine segmental and global longitudinal strain. After entering the cuff blood pressure value, the software automatically generated the LVPSL curve, GLS, and myocardial work parameters. The absolute value of GLS was recorded and the global myocardial work parameters included global work index (GWI), global constructive work (GCW), global wasted work (GWW), and global work efficiency (GWE). GWI is equivalent to the area of the PSL, GCW is a sign of active contraction of the left ventricular myocardium, GWW is a sign of energy loss, and GWE is the percentage of GCW in the sum of GCW and GWW (Figure 2).

### Cardiac Magnetic Resonance Imaging

A Siemens MAGNETOM Skyra 3.0 MR scanner (Magnetom Symphony, Siemens Medical Solutions, Erlanger, Germany), an 18-channel phased array coil for heart, and chest lead ECG gating technology were used for CMRI. The short- and long-axis images of the left ventricle were collected using a balance steady-state free procession sequence with the following parameters: TR, 3.3 ms; TE, 1.43 ms; FOV, 340 × 340 mm; matrix, 208 × 166; layer thickness, 8 mm; layer spacing, 2 mm; reversal angle, 80°; and dynamic breath-hold scanning, one cardiac cycle. A total of 25 images were collected.

For LGE image acquisition, a high-pressure syringe was used for elbow vein bolus injection of gadolinium (gadoterate meglumine, Dotarem, Guerbet, Aulnay-sous-Bois, France; 0.15 mmol/kg; flow rate of 4 mL/s). Segmented inversion was adopted to restore the gradient echo sequence. The collected short-axis images of first pass perfusion and delay period had the following parameters: layer thickness, 8 mm; TR, 6.1 ms; TE, 2.9 ms; and reversal angle, 25°. A proper reversal time was chosen to suppress normal myocardial signals.

### LV Function and LGE Analysis via Cardiac Magnetic Resonance Imaging

**Figure 3:**
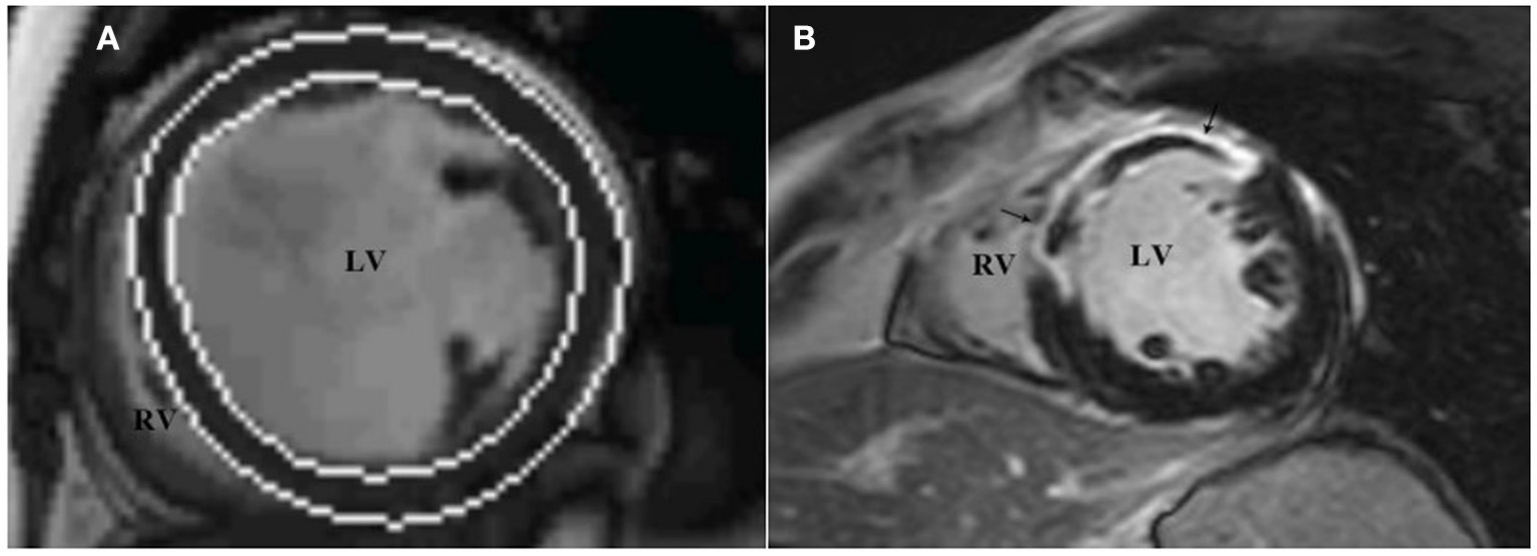
LV function and LGE analysis via cardiac magnetic resonance imaging. The analysis software automatically tracks the left ventricular endocardium and epicardium to measure left ventricular volume **(A)**, and assesses the presence and distribution of left ventricular LGE through short-axis images **(B)**, which show high signal intensity in the middle segment of the left ventricular septum, anterior wall. LGE, late gadolinium enhancement.

All images were reevaluated by two experienced observers, and all clinical data were blinded for analysis. By using Siemens Argus post-processing software (Argus software, Siemens Healthcare Erlangen, Germany), the left ventricular endocardium and epicardium were automatically tracked and contoured on the image (Figure 3A). The left ventricular cavity contained trabecular and papillary muscles. The recognition errors were corrected manually. Left ventricular end diastolic volume (LVEDV), left ventricular end systolic volume (LVESV) and left ventricular mass (LVM) were measured, and LVEF was calculated:

(1) $$\begin{array}{r} \text{LVEF}=\left(\text{LVEDV}-\text{LVESV}\right)/\text{LVEDV}. \end{array}$$

LVEDV, LVESV, and LVM were corrected as indices using BSA.

In accordance with the recommendation of the American Heart Association in 2002 (15), the left ventricular myocardium was divided into 17 segments, and the presence and distribution of LGE were evaluated using short-axis images. LGE (+) was defined as the myocardial signal at the LGE enhancement site higher than the 5SD threshold of the average signal intensity of the distal normal myocardium (Figure 3B). According to the CMRI data, the studied population was divided into two groups: patients without LGE (LGE-) and patients with LGE (LGE+).

### Biochemical Evaluation

Blood samples of patients were drawn to measure the level of N-terminal pro-brain natriuretic peptide (NT-proBNP). Analysis was conducted in the clinical laboratory.

### Statistical Analysis

Continuous variables were expressed by mean ± standard deviation when the variables obeyed normal distribution; otherwise, the median (quartile) was used. Categorical variables are presented as number (%). Baseline characteristics among patients with and without LGE were compared by chi-square or Fisher's exact test (categorical variables), and independent sample *t*-test or Wilcoxon signed rank test (continuous variables) as appropriate. To investigate the associations between variables and LV myocardial fibrosis, univariate and multivariate logistic regression analyses were performed. Correlations between independent variables were examined by Pearson correlation coefficients. For collinearity reasons (Pearson's coefficient > 0.6), several multivariate logistic regression models were built. A value of *P* < 0.05 was considered statistically significant.

Receiver operating characteristics (ROC) curve analyses were used to determine optimal cutoff points for variables in predicting LV myocardial fibrosis, and to calculate the area under the curve (AUC), sensitivity, specificity and accuracy. Intra-observer variability and inter-observer variability of myocardial work indices were assessed in 20 patients and tested using intraclass correlation coefficients (ICCs).

All statistical analyses were performed using a standard statistical software program (SPSS Version 20.0, IBM, Chicago, IL, USA).

## Results

### Study Population and Clinical Characteristics

As depicted in Figures 1, 101 patients were enrolled, 57 patients were included in the final analysis. They were divided in two groups: the LGE+ group (*n* = 32/57, 56.1%) and LGE - group (*n* = 25/57, 43.9%).

Baseline characteristics are presented in Table 1. Medications and electrocardiographic characteristics are shown in Supplementary Table 1. In the overall population (*n* = 57), mean age was 43.9 ± 12.7 years, 45 (78.95%) patients were males. QRS duration 108 (99.50, 130.50) ms, 19 (33.33%) patients had wide QRS (duration ≥120 ms). Eight (14.04%) patients had left bundle branch block, The LGE+ group presented with increased age and the difference between the two groups was statistically significant (*P* < 0.001). There were no statistically significant differences in sex, BSA, heart rate, blood pressure, NYHA functional class, NT-proBNP, concomitant diseases, medications, or electrocardiographic parameters between the two groups (*P* > 0.05).

**Table 1:** Baseline characteristics of the study population and according to presence of LGE.

**Clinical characteristics**	**All patients *n* = 57**	**LGE+ group *n* = 32**	**LGE– group *n* = 25**	***P-*value**
Age, years	43.9 ± 12.7	47.5 ± 12.5	39.4 ± 11.5	0.02
Male, *n* (%)	45 (78.95)	25 (78.12)	20 (80.00)	0.86
BSA, m^2^	2.00 ± 0.19	2.02 ± 0.20	1.98 ± 0.20	0.54
Heart rate, bpm	74.26 ± 5.42	73.53 ± 5.56	75.20 ± 5.20	0.25
Systolic blood pressure, mm Hg	118.24 ± 12.46	120.44 ± 12.95	115.44 ± 11.43	0.13
Diastolic blood pressure, mm Hg	75.47 ± 8.88	75.47 ± 9.31	75.48 ± 8.49	0.99
Diabetes mellitus, *n* (%)	12 (21.05)	6 (18.75)	6 (24.00)	0.63
Dyslipidemia, *n* (%)	21 (36.84)	12 (37.50)	9 (36.00)	0.91
NYHA functional class II/III/IV, *n*	11/25/21	6/12/14	5/13/7	0.40
NT-proBNP, pg/mL	1560 (764, 2826.5)	1672 (985.75, 3173.00)	764 (374.50, 1515.00)	0.23

*BSA, body surface area; NYHA, New York Heart Association; NT-proBNP, N-terminal pro-Brain Natriuretic Peptide. Data are expressed as mean ± SD, median (IQR), or number (percentage)*.

### Parameters Measured by Echocardiography and Cardiac Magnetic Resonance Imaging

As shown in Tables 2, 3, the LGE+ group presented with increased LVESV index (*P* < 0.05) and reduced LVEF_CMRI_, GLS, GWI, GCW, and GWE. The differences between the two groups were statistically significant (*P* < 0.001). There were no statistically significant differences in LAV index, LVEF_Simpson_, E/e′, or LVM index between the two groups (*P*> 0.05).

**Table 2:** Echocardiography parameters for the overall population and according to presence of LGE.

**Variables**	**All patients *n* = 57**	**LGE+ group *n* = 32**	**LGE– group *n* = 25**	***P-*value**
LAV index, mL/m^2^	40.65 ± 2.35	41.14 ± 2.17	40.02 ± 2.45	0.07
E/e′	16.15 ± 3.26	16.70 ± 2.87	15.44 ± 3.64	0.15
LVEF_Simpson_, %	29.32 ± 7.45	27.09 ± 5.59	29.76 ± 4.88	0.07
GWI, mm Hg%	633.61 ± 257.74	507.78 ± 172.75	794.68 ± 261.38	<0.001
GCW, mm Hg%	817.67 ± 323.58	642.25 ± 200.43	1042.20 ± 324.45	<0.001
GWW, mm Hg%	169 (123.50, 276.50)	183 (134.00, 298.25)	141 (105.50, 219.00)	0.05
GWE, %	79.26 ± 10.13	74.22 ± 9.94	85.72 ± 5.87	<0.001
GLS, %	6 (5.00, 8.00)	5 (4.00, 6.75)	8 (5.50, 11.00)	<0.001

*LAV, left atrial volume; E/e', ratio of the peak mitral flow velocity (E peak) to the average velocity of the mitral annulus (e'); GWI, global work index; GCW, global constructive work; GWW, global wasted work; GWE, global work efficiency; GLS, global longitudinal strain; LVEF_Simpson_, left ventricular ejection fraction measured using the biplane Simpson method; LGE, late gadolinium enhancement; Data are expressed as mean ± SD, median (IQR)*.

**Table 3:** Cardiac magnetic resonance imaging parameters for the overall population and according to presence of LGE.

**Variables**	**All patients *n* = 57**	**LGE+ group *n* = 32**	**LGE– group *n* = 25**	***P-*value**
LVEDV index, mL/m^2^	157.37 ± 39.93	165.45 ± 34.80	147.04 ± 44.24	0.08
LVESV index, mL/m^2^	126.79 ± 36.66	139.36 ± 30.84	110.70 ± 37.78	0.003
LVEF_CMRI_, %	20.09 ± 7.16	15.86 ± 3.85	25.52 ± 6.79	<0.001
LVM index, g/m^2^	110.19 ± 31.45	112.49 ± 31.19	107.26 ± 32.17	0.54

*LVEDV, left ventricular end diastolic volume; LVESV, left ventricular end systolic volume; LVEF_CMRI_, cardiac magnetic resonance imaging-derived left ventricular ejection fraction; LVM, left ventricular mass; LGE, late gadolinium enhancement; Data are expressed as mean ± SD*.

### Association Between Parameters and LV Myocardial Fibrosis

The univariate logistic regression analysis showed an association between LV myocardial fibrosis and the following parameters (Table 4): Age, LVESV index, LVEF_CMRI_, GLS, GWI, GCW, and GWE. There was no significant correlation between age, LVESV index and parameters of LV function, including LVEF_CMRI_, GLS, GWI, GCW, and GWE. Considering the significant correlations among parameters of LV function, several multivariate logistic regression models with one parameter of LV function and age, and LVESV index were built (Table 5). After correcting for age and LVESV index, LVEF_CMRI_, GLS, GWI, GCW, and GWE retained independent associations with LV myocardial fibrosis.

**Table 4:** Univariate logistic regression analysis to identify the determinants of LV myocardial fibrosis at late gadolinium enhancement.

**Variables**	**OR (95% CI)**	***P-*value**
Age	1.057 (1.009~1.108)	0.002
Sex, male	0.893 (0.246~3.242)	0.86
NYHA functional class	1.380 (0.668~2.848)	0.38
NT-proBNP	1.000 (1.000~1.001)	0.19
LVESV index	1.026 (1.007~1.045)	0.01
LVEF_CMRI_, %	0.649 (0.508~0.829)	0.001
LVEF_Simpson_	0.907 (0.817~1.008)	0.07
GWI	0.994 (0.990~0.997)	0.001
GCW	0.994 (0.991~0.997)	<0.001
GWE	0.807 (0.715~0.911)	0.001
GLS	0.541 (0.376~0.779)	0.001

*OR, odds ratio; 95% CI, 95% Confidence Interval; NYHA, New York Heart Association; NT-proBNP, N-terminal pro-Brain Natriuretic Peptide; LVESV, left ventricular end systolic volume; LVEF_CMRI_, cardiac magnetic resonance imaging-derived left ventricular ejection fraction; LVEF_Simpson_, left ventricular ejection fraction measured using the biplane Simpson method; GWI, global work index; GCW, global constructive work; GWE, global work efficiency; GLS, global longitudinal strain*.

**Table 5:** Multivariate logistic regression models to predict LV myocardial fibrosis.

**Multivariate models**		**OR (95% CI)**	***P-*value**
Model 1	Age	1.073 (0.999~1.153)	0.05
	LVESV index	1.002 (0.975~1.029)	0.90
	LVEF_CMRI_	0.627 (0.465~0.844)	0.002
Model 2	Age	1.051 (0.999~1.041)	0.09
	LVESV index	1.016 (0.770~0.966)	0.15
	GLS	0.626 (0.429~0.912)	0.02
Model 3	Age	1.053 (0.993~1.117)	0.08
	LVESV index	1.019 (0.997~1.042)	0.09
	GWI	0.995 (0.992~0.999)	0.01
Model 4	Age	1.037 (0.976~1.101)	0.24
	LVESV index	1.014 (0.992~1.038)	0.21
	GCW	0.995 (0.992~0.999)	0.004
Model 5	Age	1.034 (0.974~1.096)	0.27
	LVESV index	1.016 (0.994~1.038)	0.15
	GWE	0.837 (0.737~0.951)	0.01

*OR, odds ratio; 95% CI, 95% Confidence Interval*.

**Figure 4:**
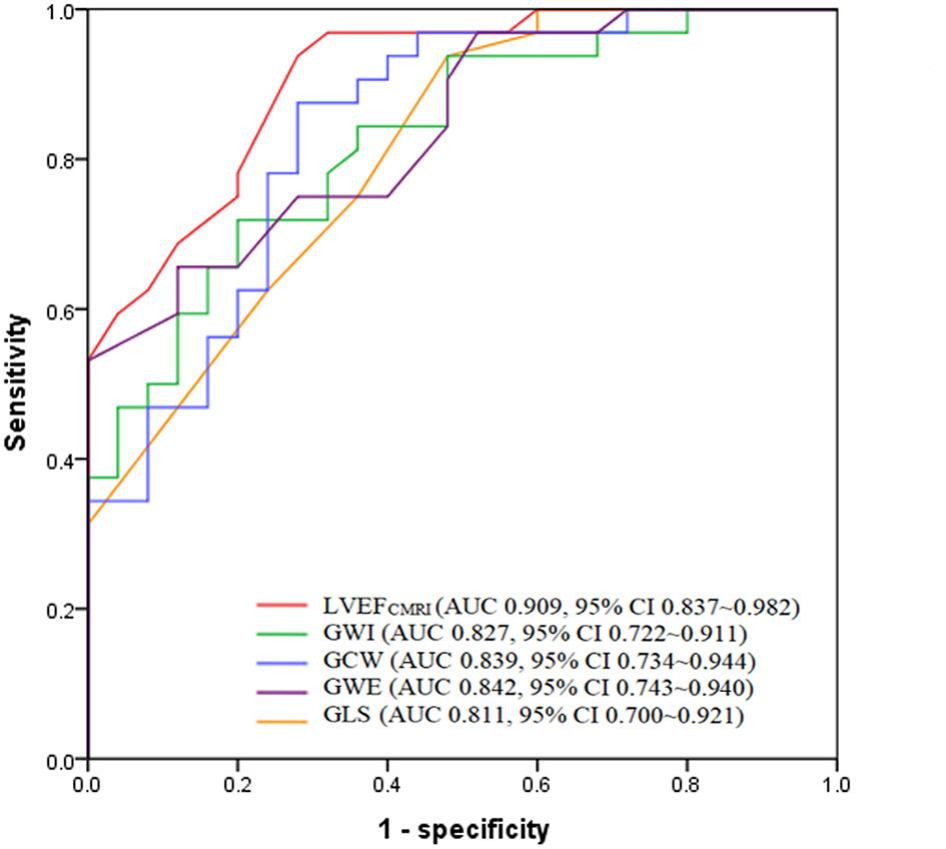
Receiver operating characteristic analysis of LVEF_CMRI_, GWI, GCW, GWE, and GLS for predicting LV myocardial fibrosis. LVEF_CMRI_, cardiac magnetic resonance imaging-derived left ventricular ejection fraction; GWI, Global work index; GCW, Global constructive work; GWE, Global work efficiency; GLS, Global longitudinal strain.

According to ROC analysis, the AUC of LVEF_CMRI_ was larger than that of GLS, GWI, GCW, and GWE, as shown in Figure 4. According to the Youden index, the cutoff point of LVEF_CMRI_ was 20.50%, the corresponding sensitivity was 93.80%, the specificity was 72.00%, and the accuracy was 84.21%. The cutoff point of GWE was 78.50%, the corresponding sensitivity was 65.60%, the specificity was 88.00%, and the accuracy was 75.43%. The cutoff points and corresponding sensitivity, specificity, accuracy of GWI, GCW, and GLS are shown in Supplementary Table 2, LVEF_CMRI_, GCW showed larger AUC and higher accuracy, sensitivity, and specificity than GLS, the accuracy of predicting LV myocardial fibrosis ranged from high to low as: LVEF_CMRI_, GCW, GWE, GWI, and GLS.

### Reproducibility of Myocardial Work Indices

Intra-observer variability and inter-observer variability of GWI, GCW, GWW, and GWE are presented in Supplementary Table 3. Intra-observer variability and inter-observer variability were the lowest for GWI, represented by the highest ICCs, ICCs were 0.910 for inter-observer and 0.961 for intra-observer measurements.

## Discussion

This study was the first to analyze the correlation between myocardial work indices and myocardial fibrosis in patients with DCM. We found that LVEF_CMRI_, GWI, GCW, GWE, and GLS remained significant predictors of LV myocardial fibrosis. LVEF_CMRI_, and GCW showed larger AUC and higher accuracy, sensitivity, and specificity than GLS, the accuracy of predicting LV myocardial fibrosis ranged from high to low as: LVEF_CMRI_, GCW, GWE, GWI, and GLS.

### Prognostic Value of Myocardial Fibrosis in Patients With DCM

The pathological bases of DCM are myocardial degeneration, atrophy, and fibrosis. Myocardial fibrosis is associated with increased risk for mortality, arrhythmia events, hospitalizations, and sudden death, therefore, detection of myocardial fibrosis has important clinical value in evaluating prognosis (16). CMRI with LGE detects myocardial fibrosis with high sensitivity and specificity. Presence of LGE is significantly associated with adverse outcome and is recommended for risk stratification of DCM patients (17). Absence of LGE is independently correlated with LV reverse remodeling, irrespective of the severity of LV dilatation and dysfunction (18). Progressive myocardial fibrosis is associated with a more than 3-fold higher risk for mortality and heart failure outcomes (19).

The association between non-invasive myocardial work indices and LV myocardial fibrosis in patients with DCM has not yet been reported. In our study, we found that global myocardial work indices, including GWI, GCW, GWE, remained significant predictors of LGE in DCM patients, indicating that these parameters may be potential surrogate markers for the detection of fibrosis when CMRI is contraindicated (significant chronic renal disease, incompatible metallic devices, or claustrophobia).

### Association Between Myocardial Dysfunction and Myocardial Fibrosis in Patients With DCM

Myocardial fibrosis leads to the decrease in ability of myocardial movement and deformation, manifested as impairment of myocardial global systolic function (4, 5, 20). Myocardial dysfunction is closely related to fibrosis in DCM patients. Previous studies have found that LVEF is significantly negatively correlated with myocardial fibrosis in DCM patients (21), while progressive fibrosis is associated with minimal change in LVEF (19), and GLS in the LGE+ group is lower than that in LGE- group (22). In this study, the LGE+ group presented with reduced GWI, GCW, GWE, GLS, and LVEF_CMRI_. These findings confirmed the association between myocardial dysfunction and fibrosis.

Additionally, in our study, multivariate logistic regression analysis showed that GWI, GCW, GWE, and GLS were independent predictors for myocardial fibrosis in addition to LVEF_CMRI._ LVEF_CMRI_, and GCW showed larger AUC and higher accuracy, sensitivity, and specificity than GLS, the accuracy of predicting LV myocardial fibrosis ranged from high to low as: LVEF_CMRI_, GCW, GWE, GWI, and GLS. Therefore, apart from CMRI, LV myocardial work indices may provide further insights into LV myocardial fibrosis in patients with DCM. The optimal cutoff points of LVEF_CMRI_, GWI, GCW, GWE, and GLS might provide valuable information. Galli et al. (23) found that GCW was significantly correlated with myocardial fibrosis in patients with hypertrophic cardiomyopathy. In future work, the application value of LV myocardial work indices in other cardiovascular diseases will be further explored to verify its clinical value.

Wide QRS (duration ≥120 ms) on the ECG represents intraventricular conduction delay. In the study, there was no statistically significant difference in QRS duration between LGE- and LGE+ patients, indicating no significant association between QRS duration and LV myocardial fibrosis. A previous study (24) found that the combination of wide QRS and LGE can provide additional risk stratification compared with LGE status alone in DCM patients.

### Limitations

This study was conducted in a single center with a small number of samples and needs to be expanded for further study. A previous study (25) found that LV midwall LGE showed an excellent predictive value in identifying high-risk DCM patients, but the correlation between the localization of LGE (sub-endocardial, mid-segment, sub-epicardial) and myocardial work parameters in this study were not explored. In addition, the accuracy of myocardial work indices in the evaluation of therapeutic response to medications were not analyzed. Further studies with extended follow-up are needed to verify the results of this study.

## Conclusion

LVEF_CMRI_, GWI, GCW, GWE, and GLS remained significant predictors of LV myocardial fibrosis. LVEF_CMRI_, and GCW appeared to better predict LV myocardial fibrosis compared with GLS. LV myocardial work indices may be potential surrogate markers for the detection of fibrosis in addition to LVEF_CMRI_.

## Data Availability Statement

The original contributions presented in the study are included in the article/Supplementary Material, further inquiries can be directed to the corresponding author/s.

## Ethics Statement

The studies involving human participants were reviewed and approved by the ethics committee of Fuwai Central China Cardiovascular Hospital. The patients/participants provided their written informed consent to participate in this study. Written informed consent was obtained from the individual(s) for the publication of any potentially identifiable images or data included in this article.

## Author Contributions

CC and LL designed the study, analyzed data, wrote the manuscript, and reviewed and edited the manuscript. YLi, YLiu, DH, YH, YW, and LM performed this study. All authors read and approved the manuscript.

## Conflict of Interest

The authors declare that the research was conducted in the absence of any commercial or financial relationships that could be construed as a potential conflict of interest.

## Publisher's Note

All claims expressed in this article are solely those of the authors and do not necessarily represent those of their affiliated organizations, or those of the publisher, the editors and the reviewers. Any product that may be evaluated in this article, or claim that may be made by its manufacturer, is not guaranteed or endorsed by the publisher.
