# Supplementary material for: Association Between Echocardiographic Non-invasive Myocardial Work Indices and Myocardial Fibrosis in Patients With Dilated Cardiomyopathy
Source: Front Cardiovasc Med. 2021 Aug 16;8:704251. doi: 10.3389/fcvm.2021.704251 (PMC8415625; doi:10.3389/fcvm.2021.704251)
Supplement: Supplementary file 1 [file Data_Sheet_1.doc]

**TABLE 1 Medications and electrocardiographic characteristics for the overall population and according to presence of LGE**

|  | **All patients**  n=57 | **LGE+ group**  n=32 | **LGE- group**  n=25 | ***P* value** |
| --- | --- | --- | --- | --- |
| **Medications** |  |  |  |  |
| Beta-blockers, n (%) | 44 (77.19) | 24 (75.00) | 20 (80.00) | 0.67 |
| ACEIs or ARBs, n (%) | 45 (78.95) | 25 (78.12) | 20 (80.00) | 0.86 |
| Statins, n (%) | 21 (36.84) | 12 (37.50) | 9 (36.00) | 0.91 |
| Antiplatelet therapy, n (%) | 38 (66.67) | 20 (62.50) | 18 (72.00) | 0.45 |
| Diuretics, n (%) | 40 (70.18) | 22 (68.75) | 18 (72.00) | 0.79 |
| Digitalis, n (%) | 14 (24.56) | 8 (25.00) | 6 (24.00) | 0.93 |
| **Electrocardiogram** |  |  |  |  |
| Left bundle branch block, n (%) | 8 (14.04) | 5 (15.63) | 3 (12.00) | 0.50 |
| QRS duration, ms | 108 (99.50, 130.50) | 112 (101.25, 129.25) | 104 (94, 131) | 0.28 |
| Wide QRS, n (%) | 19 (33.33) | 10 (31.25) | 9 (36) | 0.71 |

*ACEI, angiotensin converting enzyme inhibitor; ARB, angiotensin receptor blocker; LGE, late gadolinium enhancement,; Data are expressed as number (percentage) or median (IQR)*

**TABLE 2 Receiver operating characteristic analysis of parameters for predicting LV myocardial fibrosis**

| **Variables** | **AUC (95% CI)** | **Cutoff points** | **Sensitivity (%)** | **Specificity (%)** | **Accuracy**  **(%)** | ***P* value** |
| --- | --- | --- | --- | --- | --- | --- |
| LVEF_CMRI_, % | 0.909 (0.837~0.982) | 20.50 | 93.80 | 72.00 | 84.21 | ＜0.001 |
| GWI, mm Hg% | 0.827 (0.722~0.911) | 573.50 | 71.90 | 80.00 | 71.93 | ＜0.001 |
| GCW, mm Hg% | 0.839 (0.734~0.944) | 814.00 | 87.50 | 72.00 | 80.70 | ＜0.001 |
| GWE, % | 0.842  (0.743~0.940) | 78.50 | 65.60 | 88.00 | 75.43 | ＜0.001 |
| GLS, % | 0.811 (0.700~0.921) | 6.50 | 75.00 | 64.00 | 70.17 | ＜0.001 |

*95% CI, 95% Confidence Interval; LVEF_CMRI_, cardiac magnetic resonance imaging-derived left ventricular ejection fraction; GWI, global work index; GCW, global constructive work; GWE, global work efficiency; GLS, global longitudinal strain*

**TABLE 3** Reproducibility inter- and intra-observer agreement of myocardial work

Indices

| **Parameters** | **Inter-observer** | | **Intra-observer** | |
| --- | --- | --- | --- | --- |
|  | **ICC** | **95% CI** | **ICC** | **95% CI** |
| GWI, mm Hg% | 0.910 | 0.788~0.963 | 0.961 | 0.905~0.984 |
| GCW, mm Hg% | 0.896 | 0.736~0.959 | 0.945 | 0.867~0.978 |
| GWW, mm Hg% | 0.868 | 0.664~0,948 | 0.902 | 0.753~0.961 |
| GWE, % | 0.877 | 0.716~0.950 | 0.929 | 0.833~0.971 |

*GWI, global work index; GCW, global constructive work; GWW, global wasted work; GWE; ICC, intraclass correlation coefficients*
